# A Robust Solution to Variational Importance Sampling of Minimum Variance

**DOI:** 10.3390/e22121405

**Published:** 2020-12-12

**Authors:** Jerónimo Hernández-González, Jesús Cerquides

**Affiliations:** 1 Serra Húnter Fellow at Department of Mathematics and Computer Science, University of Barcelona, 08007 Barcelona, Spain; 2 Artificial Intelligence Research Institute (IIIA-CSIC), 08193 Bellaterra, Spain; cerquide@iiia.csic.es

**Keywords:** importance sampling, minimum variance unbiased estimator, Rényi divergence, variational inference, fully factorized family

## Abstract

Importance sampling is a Monte Carlo method where samples are obtained from an alternative proposal distribution. This can be used to focus the sampling process in the relevant parts of space, thus reducing the variance. Selecting the proposal that leads to the minimum variance can be formulated as an optimization problem and solved, for instance, by the use of a variational approach. Variational inference selects, from a given family, the distribution which minimizes the divergence to the distribution of interest. The Rényi projection of order 2 leads to the importance sampling estimator of minimum variance, but its computation is very costly. In this study with discrete distributions that factorize over probabilistic graphical models, we propose and evaluate an approximate projection method onto fully factored distributions. As a result of our evaluation it becomes apparent that a proposal distribution mixing the information projection with the approximate Rényi projection of order 2 could be interesting from a practical perspective.

## 1. Introduction

In many different fields, there exists a need for efficient and unbiased estimators for complex expectations. For example, in Bayesian statistics or in statistical physics, one can usually come across expectations of various quantities with respect to complex distributions which need to be computed. In this context, analytical solutions might not be available due to its computational complexity, among other issues. In those cases, we can resort to approximate estimation. Monte Carlo methods are a very popular sampling-based strategy to this end, and, specifically, importance sampling is a well-studied solution for variance reduction.

Importance sampling uses a probability distribution, called the proposal distribution, alternative to the distribution of interest, to obtain samples from. A wisely-selected proposal distribution would help to reduce the variance of the estimator. The characteristics of the optimal proposal distribution have largely been studied [1,2]. In our preliminary study [3], we showed that the optimal proposal distribution is also the one that minimizes the Rényi divergence with r=2 and presented an algorithm for computing the Rényi projection of a (unfactored) distribution onto the family of fully factored probability distributions.

However, due to different reasonable practical issues, this theoretical best proposal might malfunction in daily practice. For example, if the proposal distribution is selected from an easy-to-sample family of distributions, the chosen density might be far away from the theoretical optimum. More importantly, if the distribution is sampled only a reasonable number of times, the optimum distribution may show a larger observed variance than other theoretically worse solutions. Distributions with a very improbable piece of mass which introduces a very large variance are usually discarded although in limited realistic scenarios they might be really competitive.

In this paper, we extend our preliminary study [3] by proposing a projection algorithm for graphical models that takes into account the factorization of the distribution we are projecting so as to increase its efficiency. We present a competitive approximate projection which heuristically considers the subset of factors that minimizes entropy. This solution suffers from the above referred practical issues too. We show that the use of a mixture of distributions obtained from the Rényi (R) and the information (I) projections ([4], Chpt. 8), a mode-matching projection usually with large theoretical variance, is useful in practice. A small contribution of the component obtained from the I-projection is enough to maintain the empirical variance controlled. Indeed, the theoretical variance of the resulting mixture is bounded through the use of control variates [5]. To the best of our knowledge, this is the first work that proposes mixing different projections of a very same distribution to build a highly efficient proposal distribution for importance sampling.

The rest of the paper is organized as follows. First of all, the background concepts are presented. In Section 3 we present our approximate Rényi projection as a competitive proposal for importance sampling. Then, we discuss a practical issue of the theoretical minimum-variance estimator and present, in Section 5, a solution based on proposal mixture distributions. Then, our final proposal is empirically tested. The paper finishes visiting several related works and drawing conclusions and future work.

## 2. Background

The techniques presented in this work are aimed to solve a type of problems where the objective is to compute an expectation such as,

(1) ℓ=E[f(X)]=∫f(x)ρ(x)dx

where X is a random vector which follows a continuous probability distribution with density function ρ and f(x) is a real-valued function usually known as the performance function. Frequently in these complex scenarios, for different reasons, the integral in Equation (1) cannot be solved analytically, and we need to resort to approximate numerical integration.

Sampling methods aim to approximate *ℓ* by evaluating and averaging the performance function f at a sufficiently large set of randomly sampled points ${\left\{x_{i}\right\}}_{i=1}^{N}$. Monte Carlo (MC) is probably the most popular and simple approach. It obtains the random sample ${\left\{x_{i}\right\}}_{i=1}^{N}$ from ρ and estimates *ℓ* as the sample mean of their evaluation in f:

(2) $${\hat{\ell }}_{MC}=N^{-1}\sum_{i=1}^{N}f(x_{i}).$$

where ${\hat{\ell }}_{MC}$ is an unbiased estimator of *ℓ*, in the sense that $E({\hat{\ell }}_{MC})=\ell$. Moreover, by the law of large numbers, ${\hat{\ell }}_{MC}$ converges to *ℓ* with probability 1 as N→∞.

In spite of its popularity, MC presents a few drawbacks that prevent its effective use in mainly two different scenarios: (i) when the probability distribution ρ is highly complex and independent points cannot be easily generated from it, and (ii) when the values of the function f(x) vary vastly between regions.

Importance sampling (IS) is a well-known alternative that deals with both mentioned MC drawbacks. In this case, a different distribution *q* is considered such that:

$$\ell =E\left[f\left(X\right)\right]=\int f(x)\frac{\rho (x)}{q(x)}q\left(x\right)dx$$

Posing the integral in this form reveals the IS approach. A set of points ${\left\{x_{i}\right\}}_{i=1}^{N}$ are sampled from *q*, which is known as the proposal distribution. In turn, *ℓ* is estimated as the weighted sample mean of the points’ evaluations in f:

(3) $${\hat{\ell }}_{IS}=N^{-1}\sum_{i=1}^{N}f(x_{i})\frac{\rho (x_{i})}{q(x_{i})}.$$

where the quotient $\rho (x_{i})/q(x_{i})$ is the weight for samples $x_{i}$, which adjusts the contribution of $f(x_{i})\;\mathrm{to the distribution}\;\rho \;\mathrm{while the influence of}\;q\;\mathrm{is compensated}.$

The proposal distribution *q* in IS is usually selected from an easy to sample family of distributions to deal with the first difficult scenario described above. To face the second scenario, *q* may be selected to reduce the variance. In this way, it is well-known that the optimal proposal distribution *q* is proportional to |f·ρ| [1,2].

Variational inference (VI) provides a set of tools to find in a simpler family of distributions the one which is most similar to a (usually very complex) distribution *p* of interest, that is, to project the distribution *p* onto a family of our choice, Q:

$$q^{*}=proj_{Q}\left[p\right]=\underset{q\in Q}{arg min}D\left(p||q\right)$$

Then, one can use the projected distribution $q^{*}$ selected from family Q to approximate the original distribution. It is stated as an optimization problem where we want to find the member of family Q which is closer to *p* regarding some divergence, D(·||·) (A detailed introduction to divergences can be found in [6]). A popular choice for Q is the family of fully factorized distributions, which places a strong assumption of independence between variables:

$$q\left(x\right)=\prod_{i}q_{i}\left(x_{i}\right)$$

Regarding the divergence, the reverse (Note that KL is not symmetric.) Kullback–Leibler (KL) divergence

(4) $$KL\left(q||p\right)=\int_{x}q\left(x\right)\log\frac{q(x)}{p(x)}dx+\int_{x}\left(p(x)-q(x)\right)dx$$

where the last term is a correction factor so that it applies to unnormalized distributions [7], leads to the formulation of the optimization problem as follows,

$$q^{*}=\underset{q\in Q}{arg min}KL\left(q||p\right)$$

This minimization problem, known as the information (I) projection, has an efficient solving algorithm for factored distributions *p* and the fully factorized family Q, the Mean Field (MF) algorithm ([4], Chpt. 8). For each variable $X_{i}\in X$, the updating function

(5) $$q_{i}\left(x_{i}\right)\propto \exp\left(\sum_{p_{j}:X_{i}\in d\left(p_{j}\right)}\int_{z}q\left(z,x_{i}\right)\log p_{j}\left(z,x_{i}\right)dz\right)$$

is iteratively applied until convergence, where $z\in \Omega_{X-X_{i}}$, $p_{j}$ are factors of the distribution *p*, and $d(p_{j})$ is a function that returns the variables in the scope of $p_{j}$.

Variational Importance Sampling (VIS) proposes to use variational inference techniques to find the proposal distribution to perform importance sampling. In our preliminary paper [3], we explored the use of VI techniques to find the proposal distribution of minimum variance for IS as $q=proj_{Q}\left[|f\cdot \rho |\right]$. The use of the I-projection was shown to be inefficient in practice due to its zero-forcing behavior [8] which leads to fitting *q* to a mode of |f·ρ|. We proposed to use the Rényi divergence as an alternative.

The Rényi divergence is in fact a family of divergences parametrized by *r*:

(6) $$D\left(p||q\right)=Renyi_{r}\left(p||q\right)=\frac{1}{r-1}\log\int_{x}p^{r}\left(x\right)q^{1-r}\left(x\right)dx$$

The projection of p=|f·ρ| with respect to the Rényi divergence with r=2 leads to the *q* of minimum variance for IS. We developed the algorithm for the Rényi (R) projection of unfactored distributions [3], which simply iterates

(7) $$q_{i}\left(x_{i}\right)\propto \sqrt{\int \frac{p^{2}\left(x_{i},x_{-i}\right)}{q_{-i}\left(x_{-i}\right)}dx_{-i}}$$

for all $X_{i}$ until convergence, where $q_{-i}=\prod_{j\neq i}q_{j}$. However, this method does not scale well with the size of X and easily becomes unfeasible in realistic scenarios as it requires to sum over all the possible configurations of x (only $x_{i}$ is fixed).

In this paper, we present an efficient method for discrete factored distributions that performs IS with a proposal of minimum variance projected from p=|f·ρ|. To deal with the impracticability of Rényi projection, an approximation that heuristically limits the considered factors based on a minimum-entropy criterion is used. For the sake of simplicity, in the rest of the paper the family of fully factored distributions Q is considered to project onto.

## 3. An Approximation to the Rényi Projection

The main drawback for using the exact Rényi projection of Equation (7) is the marginalization of all the variables but $x_{i}$. Its computational time is exponential on the size of X. To simplify this projection so that it becomes feasible, several approaches can be taken. We propose to take advantage of the factorization of *p* to greedily select a subset of relevant factors from which to project.

Assuming the Rényi divergence definition in Equation (6), we aim to find each $q_{i}$ such that

(8) $$\begin{array}{cc} q_{i}^{*} & =\;\underset{q_{i}}{arg min}Renyi_{r}\left(p||q_{i}\cdot q_{-i}\right)\\ & \approx \;\underset{q_{i}}{arg min}Renyi_{r}\left({\tilde{p}}_{i}\left|\right|q_{i}\cdot {\left(q_{-i}\right)}^{↓d({\tilde{p}}_{i})}\right) \end{array}$$

where ${\tilde{p}}_{i}$ is the product of the subset of factors from *p* that determine the most the marginal $p(x_{i})$, and ${\left(q_{-i}\right)}^{↓d({\tilde{p}}_{i})}$ denotes the marginalization of all the variables but those in $d({\tilde{p}}_{i})$ from $q_{-i}$. Many alternatives could be considered for the selection of the relevant factors involved in ${\tilde{p}}_{i}$. As detailed below, an entropy-based criterion is considered in this paper.

To find the $q_{i}$, we need to define the Lagrangian:

(9) $$\begin{array}{cc} G(q_{i})= & \int_{x\in \Omega_{d({\tilde{p}}_{i})}}{\tilde{p}}_{i}^{r}\left(x\right)q_{i}^{1-r}\left(x\right){\left(q_{-i}^{↓d({\tilde{p}}_{i})}\left(x\right)\right)}^{1-r}dx\\ \; & -\lambda_{i}\left(\int_{y\in \Omega_{d(q_{i})}}q_{i}\left(y\right)dy-1\right) \end{array}$$

Setting to zero the derivative with respect to $q_{i}\left(y\right)$, for $Y=d(q_{i})$ and $y\in \Omega_{Y}$, $\frac{\partial G}{\partial q_{i}\left(y\right)}=0$, we obtain

$$\begin{array}{cc} 0 & =\frac{\partial \left(\int_{z,y}{\tilde{p}}_{i}^{r}\left(y,z\right)q_{i}^{1-r}\left(y\right){\left(q_{-i}^{↓d({\tilde{p}}_{i})}\left(y,z\right)\right)}^{1-r}dzdy\right)}{\partial q_{i}\left(y\right)}-\lambda_{i}\\ \; & =\left(1-r\right)q_{i}^{-r}\left(y\right)\int_{z}{\tilde{p}}_{i}^{r}\left(y,z\right){\left(q_{-i}^{↓d({\tilde{p}}_{i})}\left(y,z\right)\right)}^{1-r}dz-\lambda_{i} \end{array}$$

where $Z=d\left({\tilde{p}}_{i}\right)\backslash d\left(q_{i}\right)$ and $z\in \Omega_{Z}$, and, thus,

$$q_{i}\left(y\right)\propto {\left[\int_{z}{\tilde{p}}_{i}^{r}\left(y,z\right){\left(q_{-i}^{↓d({\tilde{p}}_{i})}\left(y,z\right)\right)}^{1-r}dz\right]}^{1/r}$$

which in the fully factorized case is

(10) $$q_{i}\left(y\right)\propto {\left[\int_{z}{\tilde{p}}_{i}^{r}\left(y,z\right)\prod_{j\in d({\tilde{p}}_{i})}q_{j}{\left(z_{j}\right)}^{1-r}dz\right]}^{1/r}$$

Up to this point, we have an algorithm for the approximate R projection if we iterate Equation (10) for all $X_{i}$ until convergence. Thus, we only have left the selection of ${\tilde{p}}_{i}$, a key matter of our approximation. We propose to select greedily a subset of factors among the ones of *p* according to their entropy. Initially, we take the factors that include $x_{i}$, ${\tilde{p}}_{i}=\prod_{j:x_{i}\in d\left(p_{j}\right)}p_{j}$. Then, sequentially, other factors are selected according to the following heuristic: the factor $p_{j}$ (not included yet, $p_{j}\notin {\tilde{p}}_{i}$, but in its Markov blanket, $d\left(p_{j}\right)\cap d\left({\tilde{p}}_{i}\right)\neq \emptyset$) which has the smallest entropy is included. That is, we consider the factors of lowest entropy because these are expected to impact the most on the marginal $p(x_{i})$. We keep selecting new factors $p_{j}$ until a number of variables $v_{max}$ is included, $|d\left({\tilde{p}}_{i}\right)|=v_{max}$. This number of variables $v_{max}$ is a free parameter of our approximate projection. We will study its effect in practice in Section 4.1. Note that, by being a greedy heuristic, this sequential procedure does not guarantee reaching an optimal solution to the problem of finding the set of most relevant factors (in terms of minimum entropy). However, this heuristic can efficiently provide working suboptimal solutions that allow the method to perform, as shown below, competitively in practice.

As mentioned above, this method takes advantage of the factorization of *p*: the finer-grained the factorization, the larger the expected performance improvement obtained. Note that this is a characteristic feature of ours and other methods which take advantage of a factored *p* distribution.

## 4. Empirical Study of VIS Performance

In this section, we aim to empirically show the performance of the proposed approximate Rényi projection when it is used as proposal distribution for importance sampling.

In this extensive set of experiments, our approach (VIS-Rh) is compared to other approaches or baselines, namely, the use of the popular I-projection (VIS-I) and the exact R projection (VIS-R). Moreover, we also implemented another approximate projection method proposed by Minka [9]. Their approach (VIS-Rm) assumes that there exists a factor $\delta_{j}$ for every $p_{j}$ ($d\left(\delta_{j}\right)=d\left(p_{j}\right)$) such that $\delta_{j}\left(x\right)=\prod_{i}\gamma_{ji}\left(x_{i}\right)$. Alternatively, there are per-variable factors $q_{i}\left(x_{i}\right)=\prod_{j:x_{i}\in d\left(\delta_{j}\right)}\gamma_{ji}\left(x_{i}\right)$. The simplification is at assuming that, when we want to update the $\delta_{j}$, the rest of the distribution is already well fitted,

$$Renyi_{r}\left(p||q\right)\approx Renyi_{r}\left(p_{j}\cdot \Delta_{-\delta_{j}}\left|\right|\delta_{j}\cdot \Delta_{-\delta_{j}}\right),$$

where $\Delta_{-\delta_{j}}=\prod_{l\neq j}\delta_{l}\left(x\right)$. By defining

$$\eta_{y}=\int_{z}\frac{p_{j}^{r}\left(z,y\right)\prod_{l\neq i\in d(p_{j})}q_{l}^{1-r}\left(z_{l}\right)}{{\left(\prod_{l\neq i:l\in d(\delta_{j})}q_{l}^{\backslash j}\left(z_{l}\right)q_{i}^{\backslash j}\left(y\right)\right)}^{-r}}dz,$$

the update of the γ factors is found to be:

(11) $$\gamma_{ji}\left(y\right)=\frac{1}{C\cdot q_{i}^{\backslash j}\left(y\right)}\eta_{y}^{1/r}$$

where *C* is the normalizing constant of $q_{i}\left(y\right)=C^{-1}\eta_{y}^{1/r}$. A detailed description can be found in Appendix A.

Note that VIS-R is only feasible in limited-size models, and thus, for the sake of comparison, we restrict ourselves to models for which the exact R projection can be obtained. We are only interested in Rényi projection of order r=2, so we fix this parameter. Whenever it is not explicitly stated, we use $v_{max}=7$ for our method. In the implementation of both approximated approaches (VIS-Rh and VIS-Rm), a damping factor ϵ=0.5 is used for the updating operations in order to benefit convergence of these heuristic methods: $s^{\left(t\right)}={\left(s^{\left(t-1\right)}\right)}^{\epsilon }\cdot {\left(s^{\prime }\right)}^{\left(1-\epsilon \right)}$ where the actual update in time *t* is a combination of the value in the previous time and the update $s^{\prime }$ given by Eqs. 10 or 11, correspondingly.

*Synthetic problems.* All the experiments are carried out with 4×4 Ising Grid models with binary variables, where unary factors are defined as $u\left(x_{i}=k\right)=\exp\left(\kappa \cdot c_{ik}\right)$ with $c_{ik}∼U\left(0,1\right)$, whereas pairwise factors are defined as $b\left(x_{i},x_{j}\right)=\exp\left(2^{-1/2}\cdot \tau \cdot B\right)$ with $B=\left|\begin{array}{cc} 0 & c_{ij}\\ c_{ij} & 0 \end{array}\right|$ and $c_{ij}∼U\left(0,1\right)$. Whereas the unary potential parameter κ=0.1 is fixed, we induce different dependence into the pairwise potentials by carefully choosing the value of the binary potential parameter τ. The higher the value of τ, the stronger the dependence between variables (specifically, different values in the two variables in the factor are favored), and thus, the higher the probability of generating peaky distributions. Specifically, for the sake of clarity, four different problems are generated and repeatedly used throughout this paper: two different grid examples are generated (using random seeds 7 and 17) with two different dependence strengths (τ=3.5 and 10). Note that, given a random seed, the difference between the problems generated with different τ is only that value, as all the $c_{ik}$ and $c_{ij}$ values are the same (generated from the same seed).

In the remaining of this section, two sets of experiments are carried out: (i) an experimental comparison of the aforementioned techniques, which includes an empirical study on the impact of the value of $v_{max}$, and (ii) a test on an extreme scenario where an exhaustive (non-sampling) method is used to pose a lower bound of the variance.

### 4.1. Experimental Comparison of Different VIS Approaches

**Figure 1:**
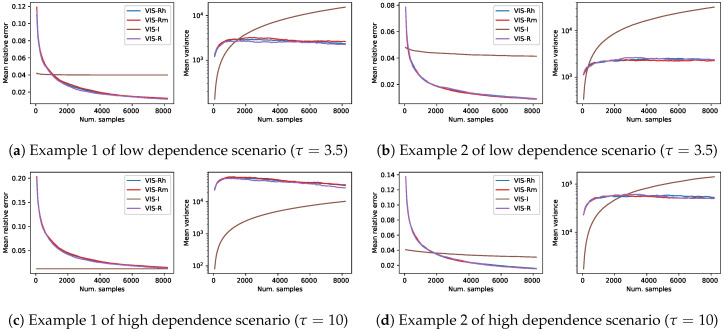
Results on four different synthetic problems (see Section 4), in terms of mean relative error and mean empirical variance (left and right plots for each subfigure, respectively), of the different VIS approaches: the I-projection (VIS-I), our approximated R projection (VIS-Rh), Minka’s approximated R projection (VIS-Rm) and the exact R projection (VIS-R). Each point in the lines is a mean over 1000 estimators with a specific number of samples in {64·k|1≤k≤128}. This is considered to cover the reasonable setups (up to 2^13^, a 12.5% of the whole sample space) for sampling based estimators.

To compare the four considered approaches in the four synthetic problems previously described, Figure 1 shows the evolution of the mean relative error and mean empirical variance ($n\cdot Var(\hat{\ell })$, in logarithmic scale) for each of the VIS methods as the number of samples used for the MC estimator grows. Each of the lines in this figure shows the mean over 1000 independent estimators. The *x*-axis shows the number of samples of each estimator, which take values on the set {64·k|1≤k≤128}. The last point of each line represents an average over 1000 estimators with $2^{13}$ samples each. This amount of samples represents about 12.5% of the whole sample space in these 4×4 Ising Grid models of binary variables. The use of approximate technique such as ours is only reasonable when there exists a considerable saving regarding, for example, computational time. Here we assume that estimators with up to $2^{13}$ samples cover all the reasonable setups. Exploring further the sample space just to obtain an approximated estimator might not make sense in real-world practice.

The performance of all the methods based on the Rényi projection (both approximated and exact versions) is similar with slight variations depending on the specific instance problem. As expected, among these three, the one that used the exact projection (VIS-R) usually showed the best performance both in terms of mean relative error and mean empirical variance. The observed behavior of VIS-I was, however, completely different: the number of samples used by the estimators almost did not affect the mean error (rather constant lines in the mean relative error figures) and the empirical variance seemed to always grow. Nevertheless, for estimators with a limited number of samples (initial points of the curves), VIS-I was consistently the best approach. In problems generated with higher dependence between variables (higher τ in pairwise factors), the overcoming of VIS-I extended to estimators with larger numbers of samples. In the case of the first model example with τ=10 (Figure 1c), not even the estimators with $2^{13}$ samples using the different Rényi projections reached the performance of VIS-I.

The main problem regarding the use of the exact R projection (VIS-R) is its high computational cost, which makes it unfeasible even for medium-size models. Thus, the time consumed by the approximate versions (VIS-Rh and VIS-Rm) should be taken into account. We have tested the four approaches (the aforementioned and VIS-I) in both 4×4 and 5×5 Ising Grid models with increasing dependence between variables (τ∈{2,3.5,5,10} for the parameter of pairwise potentials). A total of 20 randomly generated instances of each Ising Grid model example were generated. VIS-Rm showed a time consumption one order higher than that of VIS-Rh or VIS-I in both 4×4 and 5×5 grid models. When moving from 4×4 to 5×5 grid models, whereas the computation cost of these three approaches slightly increased, we observed an explosion in computational cost of VIS-R, which was much more time consuming than the rest. All the tests were performed with an Intel Core i7-7700 (3.6 GHz) with 32 GB of main memory.

**Figure 2:**
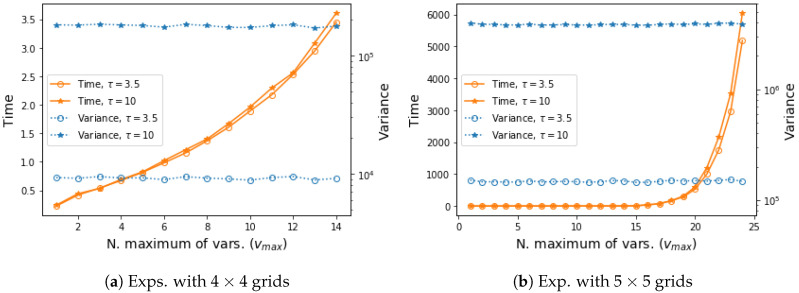
Experimental results in terms of empirical variance of VIS-Rh with different *v_max_*, as well as the time required for our approximated projection. Each figure shows the results for problem instances generated with two different dependence strengths (τ={3.5,10}). Every point is an average over 100 estimators, with 2^13^ samples each, on 20 different problem instances.

Finally, we explore the performance of our approximate approach VIS-Rh when different values for the parameter $v_{max}$ are considered. Note that as the value of $v_{max}$ tended to the number of variables of the model, $v_{max}\to v$, our approximation resembled the exact VIS-R. On the other extreme scenario, when $v_{max}=1$ only the unary factor for each specific variable was considered every time (no dependence can be captured). In Figure 2 we compare different possible values for $v_{max}$ in tests with both 4×4 and 5×5 Ising Grid models. It can be seen that the time required for projection was exponential on the $v_{max}$ values, and the empirical variance showed an almost imperceptible downwards trend.

### 4.2. Experimental Comparison versus a Deterministic Approach

The Rényi projection of order r=2 provides the distribution that minimizes the variance of the estimator. This was the motivation behind our development. However, there are decisions that might have an effect on the performance, such as the projection family of distributions considered or the simplifying assumptions of the approximate projections. Taking advantage of the fact that we are restricting ourselves to limited-size models to compare against the exact R-projection, we can observe the behavior of the methods if we use a sample size as large as the size of the whole space, $2^{4\times 4}$. In these conditions, we use an exhaustive approach to establish a clear reference to compare with. This baseline deterministic method goes thoroughly through all the points in the domain by sampling at random but without repetitions.

**Figure 3:**
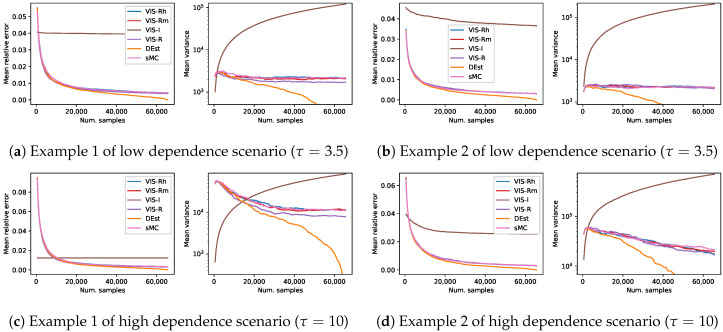
Results on four different synthetic problems (see Section 4), in terms of mean relative error and mean empirical variance (left and right plots for each subfigure, respectively), of the different VIS approaches: the I-projection (VIS-I), our approximated R projection (VIS-Rh), Minka’s approximated R projection (VIS-Rm) and the exact R projection (VIS-R). Moreover, simple Monte Carlo (MC) and an exhaustive procedure (DEst) are also included. Each point in the lines is a mean over 1000 estimators with a specific number of samples in {512·k|1≤k≤128}. This covers up to the whole sample space (2^16^).

Using the same representation than in the previous set of experiments, Figure 3 shows the results in terms of mean relative error and mean empirical variance ($n\cdot Var(\hat{\ell })$, in logarithmic scale) of the four previous approaches, the aforementioned deterministic method (DEst) and the simple MC (sMC). As before, each of the curves is the mean over 1000 estimators and shows a continuity of $2^{7}$ estimators with increasing number of samples per estimator ({512·k|1≤k≤128}). Note that these figures are somehow a zoom-out of the Figure 1: this previous figure shows the initial part of the current Figure 3. As explained before, both mean relative error of DEst and its variance tended to 0 as the number of samples got close to $2^{4\times 4}$.

The performance of all the methods based on the Rényi projection (both approximated and exact versions) showed again a similar behavior with slight variations. However, in this long term sampling experiment, the better performance of the exact R-projection approach was more perceptible (in terms of empirical variance). Moreover, the theoretically higher variance of the VIS-I approach was now observed (in the long run) for all the cases. The observed behavior of sMC in the long run was in many cases comparable to those of both VIS with approximate R projections. However, it was usually the worst approach when the estimators use a limited-size set of samples, as expected. Finally, the observed behavior of the deterministic approach, DEst, was comparable to that of the VIS approaches based on the Rényi projection in the case of estimators with a reasonable number of samples. As it went through a third of the points of the whole space, it overcame the sampling based approaches, and steadily approached zero relative error and variance afterwards. This point where DEst overcame the rest of approaches poses a clear boundary for the use of sampling based techniques in these problems: it is unnecessary to spend time looking for the best proposal distribution to sample from, if it happens that enumerating the sample space is a better approach.

### 4.3. Discussion

This extensive set of experiments has shown the strengths of our proposal. Whereas the actual R projection is unfeasible even for medium-size models, ours seems to be a competitive approximate alternative. Our approach reduces the complexity of the problem by assuming independence with respect to any variable that has not a low-entropy factor linking it to the variable of interest. Moreover, our method projects onto a single unary factor all the information regarding the corresponding variable. This is different for the other considered approximate R-projection [9], which uses multiple copies of unary factors (as many as factors with that variable in *p*) and simplifies the problem by assuming that, when projecting for a specific variable, “the distribution of the rest of the model is already fitted and the product of corresponding projections is a good approximation”. According to the previous experimental results, both approximate approaches (VIS-Rh and VIS-Rm) show a competitive behavior regarding the exact VIS-R. Moreover, the differences between VIS-Rh and VIS-Rm are hardly perceptible in terms of error and variance. However, our approach requires a lower computational effort (the difference in terms of computational time is one order of magnitude lower in favor of VIS-Rh) to obtain similar results.

The results regarding the impact of the $v_{max}$ value show that the performance is similar throughout all the experiments. This is in line with the previous discussion, where both approximations and the exact Rényi projection showed indistinguishable results. The projection time increases as with the value of $v_{max}$. The choice of $v_{max}=7$ in these experiments looks for a trade off by means of which a substantial part of the original distribution *p* is taken into account and the projection time does not increase exponentially.

A behavior that repeats all over this set of experiments is that, frequently, the empirical variance of the VIS-I approach is (extremely) lower than that of those based on the Rényi projection. However, in terms of theoretical variance, the differences are the other way around: the Rényi projection leads to the estimator with minimum theoretical variance, and that of VIS-I is (extremely) large. This poses an interesting question: how is it possible that a theoretically high-variance method shows lower empirical variance than the method that employs the theoretically optimal distribution?

**Figure 4:**
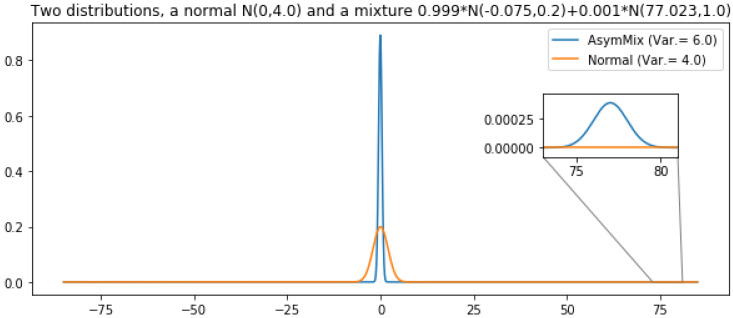
Distributions considered in the simple variance analysis. A normal distribution with variance 4 and a mixture of larger variance (6) with a large component ($\alpha_{a}=1-\alpha_{b}$, centered in −0.07, with variance 0.2) and a small component with a huge contribution to variance ($\alpha_{b}=2^{-10}$, centered in 77.02, with variance 1).

**Figure 5:**
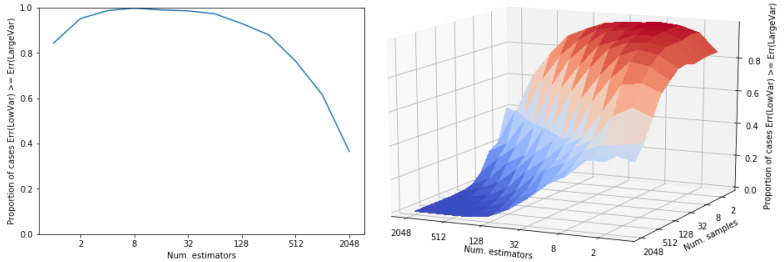
Proportion of cases (over 1000 repetitions) where the mean square error of the estimator using the lower variance distribution (normal distribution in Figure 4) is larger than that of the one using the larger variance distribution (mixture in Figure 4). On the left, the proportion for different number of estimators (a single data point sampled per estimator). On the right, the same proportion for different number of estimators and samples per estimator.

First of all, let us note that this is not an issue specific from our experimental setting. It is a general issue which is regularly observed if an otherwise low-variance distribution has a small probability mass with a very large contribution to variance. While sampling from the low mass regions with high contribution to the theoretical variance is improbable, the observed variance may be low. Let us use the following example to illustrate this situation. Let us define two distributions: (i) a normal distribution, *r*, with variance 4 and (ii) another distribution, *s*, with variance 6. Let us define this second distribution as a mixture of two normal distributions where the main component has a very low variance and there exists a second tiny component which is responsible for the large variance of the mixture (Var. = 6). See Figure 4 for a graphical description of both distributions. Note that the mass of the extreme region of distribution *s* is $2^{-10}$. We use both distributions to obtain a sampling estimator of their expected value (in both cases, E[X]=0). In Figure 5 we show the results of comparing the error of both estimators, depending on the number of estimators (and the number of samples per estimators). Both subfigures show the proportion of cases in which the error of the lower variance distribution, *r*, is larger than that of the larger variance distribution, *s*. The error of the approximation that uses distribution *s* is proportionally lower while the number of estimators do not reach $2^{10}$. That is, this depends on the mass of the extreme region of *s*. In a sampling approach, the contribution (to variance) of a region is not observed until a point is sampled from there. As the probability of sampling there increases (the number of samples is larger than the inverse of the probability of that region), its contribution to variance starts to be observed and, observed variance tends to its theoretical value. Still, many more samples are required to always observe a better performance of the theoretically lower variance estimator, *r*: it only overcomes completely the larger variance distribution *s* when the number of points (number of estimators × number of samples) is ∼$2^{20}$ (Figure 5). One might be tempted of disregarding those low mass regions but, by doing so, an unbiased high-variance estimator would be converted into a low-variance biased estimator. In the illustrative example in Figure 4 and Figure 5, the low mass region has a probability of $2^{-10}$ leading to a variance of 6, whereas in our experiments this rare events might be of the order of $2^{-30}$. Precisely, the initial advantage of VIS-I is more prominent as we induce a larger dependence between variables, that is, as the probability mass is more concentrated around one or more modes.

This discussion is really relevant because the order of samples in which VIS-I still overcomes the Rényi based approaches usually covers the reasonable scenarios for sampling. In real-world practice, we will not sample so many times, since otherwise exhaustive techniques start being competitive in terms of error and even time. In the next section, we take advantage of this type of distributions (I-projection) to propose an estimator that, as the number of samples increases, performs almost as robustly as VIS-R. We achieve this task by using a proposal distribution for IS which combines both the I and our approximate R-projections by means of a mixture distribution.

## 5. Mixture IS Approach

So far, we have considered single fully factored distributions as proposal density for IS. However, the proposal can also be a mixture distribution [5]:

$$q_{\alpha }\left(x\right)=\alpha q_{a}\left(x\right)+\left(1-\alpha \right)q_{b}\left(x\right)$$

where α∈[0,1] is the parameter that weighs the components of this simple two-component mixture.

The estimator of mixture importance sampling is similar to Equation (3), as only the proposal is changed:

(12) $${\hat{\ell }}_{MIS}=N^{-1}\sum_{i=1}^{N}f(x_{i})\frac{\rho (x_{i})}{q_{\alpha }\left(x_{i}\right)}$$

The use of mixture distributions has the interesting property that the variance of the estimator is bounded by those of the components when the same component distributions are used as control variates [5]. Control variates is a variance reduction procedure that takes advantage of the correlation between the statistic of interest and another (simple) statistic to reduce the variance of the estimator.

If the components $q_{t}$ of the mixture are distributions ($E[q_{t}]=1$), they can be used as control variates, so that the estimator (CVIS) would be as follows:

(13) $$\begin{array}{cc} {\hat{\ell }}_{CVIS}= & N^{-1}\sum_{i=1}^{N}\frac{f(x_{i})\rho \left(x_{i}\right)-\sum_{t\in \{a,b\}}\beta_{t}q_{t}\left(x_{i}\right)}{q_{\alpha }\left(x_{i}\right)}\\ & +\sum_{t\in \{a,b\}}\beta_{t} \end{array}$$

where $\beta_{a}$ and $\beta_{b}$ are the control variate coefficients of components $q_{a}$ and $q_{b}$, respectively. According to Theorem 2 of Owen and Zhou [5], the use of control variates allows for establishing an upper bound on the variance of the estimator in Equation (13):

$$\sigma_{{\hat{\ell }}_{CVIS}}^{2}\leq \min_{t\in \{a,b\}}\alpha_{t}^{-1}\sigma_{t}^{2}$$

that is, the variance of the CVIS estimator is less than the minimum variance of an IS estimator using only one component $q_{t}$ of the mixture as proposal distribution to a factor inversely proportional to the weight of the component in the mixture ($\alpha_{t}^{-1}$).

This bound holds if the control variate coefficients are close to the optimal vector β. Note that the estimation of the control variate coefficients seems to be a first step for Equation (13). However, Owen and Zhou [5] described the conditions in which this problem can be reformulated as a multiple regression

$$y_{i}={\hat{\ell }}_{CVIS}+\vec{\beta }z_{i}={\hat{\ell }}_{CVIS}+\beta_{a}z_{ai}+\beta_{b}z_{bi},$$

and, thus, the control variate coefficients, $\beta_{a}$ and $\beta_{b}$, and ${\hat{\ell }}_{CVIS}$ can be simultaneously estimated as the parameters and the intercept, respectively. In this regression setting, the dependent variable is $y_{i}=f(x_{i})\rho \left(x_{i}\right)q_{\alpha }^{-1}\left(x_{i}\right)$, and the independent variables are $z_{ti}=q_{t}\left(x_{i}\right)q_{\alpha }^{-1}\left(x_{i}\right)-1$, for t∈{a,b}. A detailed description can be found in Appendix B.

### Deterministic Mixture IS with Control Variables: Combining the Strengths of I and R Projections

We propose to use the I-projection and our approximation to the R-projection of r=2 as components of a mixture distribution for the proposal of IS.

According to Hesterberg [10], stratifying or deterministically sampling from the proposal mixture distribution reduces the empirical variance. Deterministic sampling means that each component of the mixture is separately sampled a specific number of times: in our method, $q_{a}$ is sampled Nα times and $q_{b}$, N(1−α) times (rounded to the closest integer in both cases).

## 6. Empirical Study of Mixture VIS Performance

In this section, we aim to empirically show the behavior of the mixture importance sampling with the I and our approximated R projections combined into the proposal distribution. We compare our mixture approach (VIS-Rx) to other approaches such as VIS with any of the components of the mixture (VIS-I and VIS-Rh), VIS using Minka’s approximate R projection (VIS-Rm) and the exact R projection (VIS-R). Here we follow the same experimental setting as in the previous set of experiments (Section 4). All the experiments are also carried out on the same four synthetic problems (Ising Grid model instances). With the objective of testing the behavior of the proposed method whereas as many external effects as possible are kept controlled, the following (hyper-)parameters are fixed throughout the experiments: Rényi projection of order r=2, for our approximate Rényi projection, $v_{max}=7$, a damping factor ϵ=0.5 is used for Equations (10) and (11).

In the remaining of this section, we first present an empirical study on the impact of the weight of the components of the mixture, and then a complete experimental comparison of the aforementioned techniques is carried out.

### 6.1. Empirical Study on the Importance of the Component Weight, α

**Figure 6:**
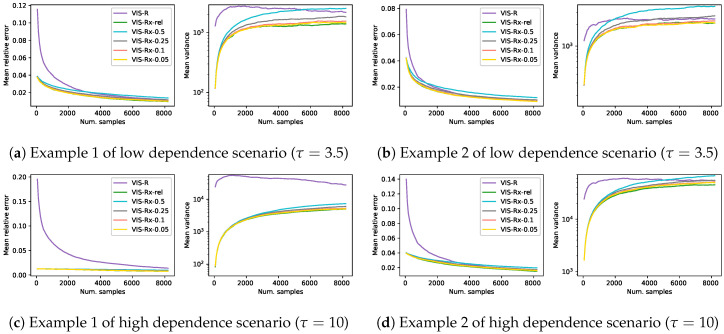
Results on four different synthetic problems (see Section 4), in terms of mean relative error and mean empirical variance (left and right plots for each subfigure, respectively), of VIS-Rx with five different a selections: constant α∈{0.5,0.25,0.1,0.05} and a decreasing *α* value (VIS-Rx-rel) relative to the number of samples. Each point in the lines is a mean over 1000 estimators with a specific number of samples in {64·k|1≤k≤128}. This is considered to cover the reasonable setups (up to 2^13^, a 12.5% of the whole sample space) for sampling based estimators.

In a mixture distribution, one of the most relevant elements is the mixture weights. In this set of experiments we aim to show some insights into the most appropriate value for the mixture weights. We test, in the experimental setup of Section 4, VIS-Rx with four different values for α∈{0.5,0.25,0.1,0.05} (α is the weight of the I-projection component, whereas 1−α is that of the R-projection) together with another approach (VIS-Rx-rel) where the weight of the I-projection is inversely proportional to the number of samples of the estimator. The VIS approach which uses the exact R-projection is also considered for comparison. Figure 6 shows the results in terms of mean relative error and mean empirical variance ($n\cdot Var(\hat{\ell })$, in logarithmic scale). Each of the lines in this figure is the mean over 1000 estimators and shows a continuity of $2^{7}$ estimators with increasing number of samples per estimator ({64·k|1≤k≤128}) (with up $2^{13}$ samples, which cover the so-called reasonable setups). It is important to remark that, in the case of VIS-Rx-rel, the mixture weight α along the corresponding line is not constant but decreasing.

The performance of VIS-Rx was similar for all the values of α. Starting from a minimum variance point, which, as observed above, was established by the I-projection, the empirical variance quickly rose to then reduce the slope and continued increasing more softly. However, smaller values of α led to reduced empirical variance. Specifically, the approach which used an α value which depended on the number of samples (the larger the sample, the smaller the proportion of I-projection points) showed consistently the best results. The behavior of VIS-R was noticeably different, starting from a larger variance and relative error points. In the case of empirical variance, after a considerable increase, it steadily decreased.

### 6.2. Experimental Comparison of Mixture Importance Sampling

**Figure 7:**
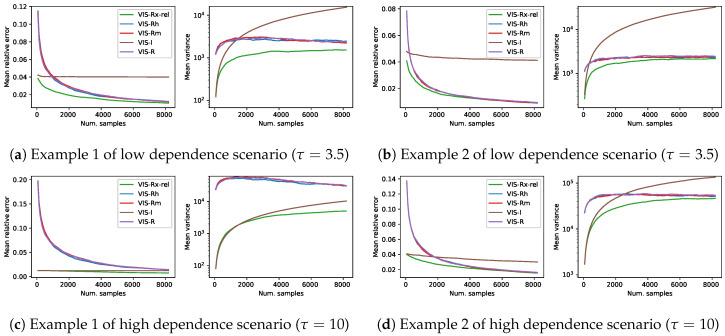
Results on four different synthetic problems (see Section 4), in terms of mean relative error and mean empirical variance (left and right plots for each subfigure, respectively), of five different VIS approaches: our mixture-based proposal (VIS-Rx-rel), those using its components (VIS-I and our VIS-Rh), Minka’s approximated R projection (VIS-Rm) and the exact R projection (VIS-R). Each point in the lines is a mean over 1000 estimators with a specific number of samples in {64·k|1≤k≤128}. This is considered to cover the reasonable setups (up to 2^13^, a 12.5% of the whole sample space) for sampling based estimators.

In this set of experiments, our mixture importance sampling approach is compared to the rest of (VIS) techniques considered in this paper. Given the results of Section 6.1, we decided to include just the results of VIS-Rx-rel as a representative of the mixture approaches. Thus, Figure 7 shows the results in terms of mean relative error and log mean empirical variance following again the experimental setup of Section 4. Our VIS-Rx-rel is compared to VIS using each of its mixture components (VIS-I and our VIS-Rh), VIS with Minka’s approximated R projection (VIS-Rm) and VIS using the exact R projection (VIS-R). Each of the lines in this figure is the mean over 1000 estimators and shows a continuity of $2^{7}$ estimators with increasing number of samples per estimator ({64·k|1≤k≤128}). In this experimental scenario with estimators limited to reasonable setups (number of samples up to 12.5% of the total space), the performance of all the methods based exclusively on the Rényi projection (both approximated and exact versions) was similar: the mean relative error was initially large and quickly decreased to obtain reasonably good estimators, whereas the empirical variance initially also increased, then stabilized and finally decreased steadily. As described in the previous section, VIS-I showed a competitive behavior in limited setups, although the mean relative error resembled that of a biased estimator and the empirical variance quickly increased as estimators of larger number of samples were used. The behavior of the mixture proposal (VIS-Rx-rel) was clearly superior to that of the rest. It started from the initial point posed by I-projection, both in terms of mean relative error and log mean empirical variance, but showed a trend more similar to that of the approaches based on the R-projection.

**Figure 8:**
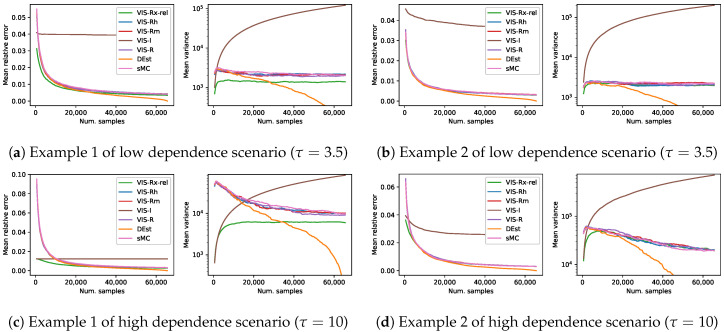
Results on four different synthetic problems (see Section 4), in terms of mean relative error and mean empirical variance (left and right plots for each subfigure, respectively), of five different VIS approaches: our mixture-based proposal (VIS-Rx-rel), those using its components (VIS-I and our VIS-Rh), Minka’s approximated R projection (VIS-Rm) and the exact R projection (VIS-R). Moreover, simple MC and an exhaustive procedure (DEst) are also included. Each point in the lines is a mean over 1000 estimators with a specific number of samples in {512·k|1≤k≤128}. This covers up to the whole sample space (2^16^).

Moreover, in order to show the whole picture, we show in Figure 8 a similar set of experiments but obtaining a number of samples up to the total size of the space. In this scenario, we again compare against the non-sampling 0-variance estimator DEst.

The performance of our mixture-based approach was better than any other method in the first grid example (with both τ=3.5, Figure 7a, and τ=10, Figure 7c), whereas in the second one VIS-Rx-rel had the best performance for estimators with limited number of samples, and it ended up showing a similar behavior to the R-projection based approaches. However, note that whenever VIS-Rx-rel was overcome (in any of the four problems in Figure 7), the best approach was the deterministic method (DEst). The performance of all the methods based on the Rényi projection (both approximated and exact versions) showed again a similar behavior, and the empirical variance of VIS-I was in line with its higher theoretical variance.

### 6.3. Discussion

Different practical ideas can be drawn from these empirical studies. Firstly, the use of a mixture of the I-projection and our approximated R-projections seems to be a reasonable choice as the proposal for IS. The I-projection places most of the probability mass in a mode of the projected distribution and leads to a theoretically high variance unbiased estimator. However, in practical limited scenarios (in terms of number of estimators/samples), the VIS-I estimator behaves as a biased low variance estimator which frequently shows a low error. The R-projection, and thus also our approximation, is a flatter distribution which leads to the theoretical minimum variance unbiased estimator. In practice, it shows a smoother behavior which involves a very competitive performance in the long run, lacking efficiency in the more limited scenarios which are considered reasonable for real-world practice. Sampling these points with a large probability mass according to the I-projection from the beginning allows VIS-Rx to show the characteristic behavior of the VIS-Rh method but starting from the competitive performance levels reached by VIS-I when the number of samples is limited. One may wonder how many samples need to be obtained from the I-projection for VIS-Rx to show this behavior. This is precisely what we tested in Section 6.1, where we studied the relevance of the mixture weight α. Our experiments show that a small proportion of samples from the I-projection is enough to attain this behavior (α=0.05). Moreover, the best result is obtained by the mixture IS approach with relative α (VIS-Rx-rel), i.e., a method where the mixture weight α depends on the number of samples of the estimator. The I-projection points to an important region that is advisable not to miss. As the number of samples of the estimator increases, the probability of missing that important region by the R-projection decreases and so does the relevance of the I-projection.

Meanwhile, the experiments of Section 6.2 show that the mixture approach is the best approach in reasonable setups. In fact, it is interesting to realize that whenever the DEst approach, a deterministic trivial solution, is the best approach, no sampling technique makes sense. Thus, we can look for the point (estimators with a specific number of samples) where DEst becomes the best approach in terms of empirical variance, for instance. Before that point, our VIS-Rx-rel is, consistently throughout all the problems, the best technique. As the number of samples used by the estimator increases, the performance of VIS-Rx-rel tends to that of the R-projection based approaches. Remember that VIS-Rx-rel uses a mixture weight α which is inversely proportional to the number of samples of the estimator. This explains the observed behavior of why VIS-Rx tends to VIS-Rh. However, a part of the experimental results (Figure 8a,c) shows that the referred convergence might require a very large number of samples (even larger than the whole domain size).

## 7. Related Work

This paper is related to different previous works in the literature.

Regarding importance sampling, the key work by Owen and Zhou [5] establishes the bases for our proposal. They propose different alternatives to improve the variance of importance sampling estimators. They expose the properties of mixture importance sampling, the use of control variates, multiple importance sampling, etc. Ours works on mixture IS with control variates. Multiple importance sampling [2], although similar in the sense of using several distributions as proposals, does not consider a mixture of them. Elvira et al. [11] presented more recently a comprehensive study of both mixture and multiple IS, showing that mixture IS is more robust than multiple IS, although it is also more expensive. They finally present a method based on multiple IS with many proposals which are in turn mixture distributions.

In information theory and variational inference, the concepts of information and momentum projections, which consider Kullback-Leibler divergence, are well established [4,8]. This classical KL divergence has been generalized and several families of divergences have been proposed, where KL is just a special case. Minka [9] took advantage of one of these families, the α-divergences, to propose a general projection method for any divergence is the family. Similarly, Regli and Silva [12] proposed a method for the α−β divergences, Wang et al. [13] for the f-divergences. In this paper, we explored the use of the Rényi family of divergences [14]. It has been previously used in the context of variational inference [15], where a new class of variational evidence lower-bounds, the variational Rényi bound, was proposed.

## 8. Conclusions

In this paper, a practical solution to importance sampling using variation inference to obtain the estimator of minimum variance is proposed. Our study considers discrete distributions, which are projected to the fully factorized family of distributions. We present a competitive approximation to the exact Rényi projection to be used as proposal distribution for IS. Note that Rényi projection of order 2 leads to the theoretical minimum variance estimator. Nevertheless, we empirically show that this theoretical minimum is overcome, in real-world set-ups, by other solutions with large theoretical variance with specific characteristics. We combine both worlds to propose the use of a mixture of the Rényi projection of order 2 and the information projection with control variates as proposal of IS. The robustness of this approach is on its ability to perform competitively across scenarios of different complexity where samples might be scarce as in many realistic situations. Indeed, the variance of this estimator is bounded by the smallest component variance to a factor determined by the mixture weights. The method has been shown to behave very competitively in a large experimental setting.

For future work, adapting this approach to project onto more expressive families of distributions—other than the fully factorized family—could be an interesting extension. Minka [9] showed that the α parameter (global) of the divergence when projecting the whole distribution does not lead to the same solution as using the same α (local) to project parts of the distribution. Something similar might be happening with Rényi’s *r* parameter. It would be interesting to study and adjust, if necessary, the local *r* parameters so that the whole projection minimizes the r=2 Rényi divergence.

## Appendix A. Minka’s Based Procedure Full Derivation

Minka [9] proposed a general method based on α-divergences for the projection of factored distributions. A factor $\delta_{j}$ for every $p_{j}$ ($d\left(\delta_{j}\right)=d\left(p_{j}\right)$) is devised. In the fully factored case, $\delta_{j}\left(x\right)=\prod_{i}\gamma_{ji}\left(x_{i}\right)$, and per-variable factors are $q_{i}\left(x_{i}\right)=\prod_{j:x_{i}\in d\left(\delta_{j}\right)}\gamma_{ji}\left(x_{i}\right)$. Thus, Q(x)≡Δ(x).

The simplification in [9] is related with the assumption of

$$Renyi_{r}\left(P||Q\right)\approx Renyi_{r}\left(p_{j}\cdot \Delta_{-\delta_{j}}\left|\right|\delta_{j}\cdot \Delta_{-\delta_{j}}\right)$$

Their algorithm was developed for the α-divergences, but it can be adapted to the Rényi divergences in Equation (6). In this case, from the previous assumption, the Rényi divergences simplify as follows,

$$\begin{array}{cc} Renyi_{r}(p_{j}\cdot \Delta_{-\delta_{j}} & \left|\right|\delta_{j}\cdot \Delta_{-\delta_{j}})\\ \; & =\int_{x,z}p_{j}^{r}\left(x\right)\delta_{j}^{1-r}\left(x\right)\Delta_{-\delta_{j}}\left(x,z\right)dxdz\\ \; & \propto \int_{x}p_{j}^{r}\left(x\right)\delta_{j}^{1-r}\left(x\right)\prod_{k\neq j}\prod_{\begin{array}{c} i:i\in d(\delta_{j})\\ \wedge i\in d(\delta_{k}) \end{array}}\gamma_{ki}\left(x_{i}\right)dx\\ \; & =\int_{x}\frac{p_{j}^{r}\left(x\right)\prod_{i\in d(p_{j})}q_{i}^{1-r}\left(x_{i}\right)}{\prod_{k\neq j}\prod_{\begin{array}{c} i:i\in d(\delta_{j})\\ \wedge i\in d(\delta_{k}) \end{array}}\gamma_{ki}^{-r}\left(x_{i}\right)}dx \end{array}$$

With this expression, the problem of finding the optimal update of the individual factors $\gamma_{ji}$ can be posed. Let us redefine $q_{i}$ as a normalized probability mass function, $q_{i}\left(x_{i}\right)=\frac{1}{s_{i}}\prod_{j:x_{i}\in d\left(\delta_{j}\right)}\gamma_{ji}\left(x_{i}\right)$. Let the Lagrangian be:

(A1) $$\begin{array}{cc} G(q_{i})= & \int_{x}\frac{p_{j}^{r}\left(x\right)\prod_{i\in d(p_{j})}q_{i}^{1-r}\left(x_{i}\right)}{\prod_{k\neq j}\prod_{\begin{array}{c} i:i\in d(\delta_{j})\\ \wedge i\in d(\delta_{k}) \end{array}}\gamma_{ki}^{-r}\left(x_{i}\right)}dx\\ \; & -\lambda_{i}\left(\int_{y\in \Omega_{d(q_{i})}}q_{i}\left(y\right)dy-1\right) \end{array}$$

Setting to zero the derivative with respect to $q_{i}\left(y\right)$, for a $y\in \Omega_{d(q_{i})}$, $\frac{\partial G}{\partial q_{i}\left(y\right)}=0$ we obtain

$$\left(1-r\right)q_{i}^{-r}\left(y\right)\int_{z}\frac{p_{j}^{r}\left(z,y\right)\prod_{l\neq i\in d(p_{j})}q_{l}^{1-r}\left(z_{l}\right)}{{\left(\prod_{l\neq i:l\in d(\delta_{j})}q_{l}^{\backslash j}\left(z_{l}\right)q_{i}^{\backslash j}\left(y\right)\right)}^{-r}}dz+\lambda_{i}=0$$

with

$$q_{i}^{\backslash j}\left(x_{i}\right)=\frac{1}{s_{i}}\prod_{k\neq j:x_{i}\in \Omega_{d(\delta_{k})}}\gamma_{ki}\left(x_{i}\right)$$

By defining

$$\eta_{y}=\int_{z}\frac{p_{j}^{r}\left(z,y\right)\prod_{l\neq i\in d(p_{j})}q_{l}^{1-r}\left(z_{l}\right)}{{\left(\prod_{l\neq i:l\in d(\delta_{j})}q_{l}^{\backslash j}\left(z_{l}\right)q_{i}^{\backslash j}\left(y\right)\right)}^{-r}}dz$$

then

(A2) $$q_{i}\left(y\right)=\frac{1}{C}\eta_{y}^{1/r}\propto \eta_{y}^{1/r}$$

Therefore, we can define the update of the γ factors as:

(A3) $$\gamma_{ji}\left(y\right)=\frac{1}{C\cdot q_{i}^{\backslash j}\left(y\right)}\eta_{y}^{1/r}$$

## Appendix B. Mixture Importance Sampling Estimator with Control Variates as a Multiple Regression

The mixture importance sampling estimator with control variates is, for this simple two component mixture, as follows,

$$\begin{array}{cc} {\hat{\ell }}_{CVIS}= & N^{-1}\sum_{i=1}^{N}\frac{f\left(x_{i}\right)p\left(x_{i}\right)-\beta_{a}q_{a}\left(x_{i}\right)-\beta_{b}q_{b}\left(x_{i}\right)}{q_{\alpha }\left(x_{i}\right)}\\ \; & +(\beta_{a}+\beta_{b}) \end{array}$$

where $\beta_{a}$ and $\beta_{b}$ are the control variate coefficients of component $q_{a}$ and $q_{b}$, respectively, and α (and 1−α) are the weights of the components in the mixture.

To solve this problem, the first step is to estimate the value of the control variate coefficients, $\left\{\beta_{a},\beta_{b}\right\}$. In this paper, we follow the idea of Owen and Zhou [5], who presented a transformation of this into a multiple regression problem and to estimate both the control variates and ${\hat{\ell }}_{CVIS}$ as the regression coefficients and the intercept, respectively. Let us re-arrange the previous equation as follows,

$$\begin{array}{cc} {\hat{\ell }}_{CVIS}= & N^{-1}\sum_{i=1}^{N}\frac{f\left(x_{i}\right)p\left(x_{i}\right)}{q_{\alpha }\left(x_{i}\right)}-\beta_{a}N^{-1}\sum_{i=1}^{N}\left(\frac{q_{a}\left(x_{i}\right)}{q_{\alpha }\left(x_{i}\right)}-1\right)\\ \; & -\beta_{b}N^{-1}\sum_{i=1}^{N}\left(\frac{q_{b}\left(x_{i}\right)}{q_{\alpha }\left(x_{i}\right)}-1\right) \end{array}$$

and

$$\begin{array}{cc} N^{-1}\sum_{i=1}^{N}\frac{f\left(x_{i}\right)p\left(x_{i}\right)}{q_{\alpha }\left(x_{i}\right)}= & {\hat{\ell }}_{CVIS}+\beta_{a}N^{-1}\sum_{i=1}^{N}\left(\frac{q_{a}\left(x_{i}\right)}{q_{\alpha }\left(x_{i}\right)}-1\right)\\ \; & +\beta_{b}N^{-1}\sum_{i=1}^{N}\left(\frac{q_{b}\left(x_{i}\right)}{q_{\alpha }\left(x_{i}\right)}-1\right) \end{array}$$

Let us define

$$y_{i}=f\left(x_{i}\right)p\left(x_{i}\right)q_{\alpha }^{-1}\left(x_{i}\right),$$

$$z_{ai}=q_{a}\left(x_{i}\right)q_{\alpha }^{-1}\left(x_{i}\right)-1,\;\mathrm{and}\;z_{bi}=q_{b}\left(x_{i}\right)q_{\alpha }^{-1}\left(x_{i}\right)-1.$$

We can express it as

$$y_{i}={\hat{\ell }}_{CVIS}+\vec{\beta }z_{i}$$

Note that this is the same definition of a multiple regression Z as random independent variables and Y as a dependent variable.

## References

[1] Kahn H., Marshall A.W. (1953). Methods of Reducing Sample Size in Monte Carlo Computations. J. Oper. Res. Soc. Am..

[2] Owen A.B. (2013). Importance Sampling. Monte Carlo Theory, Methods and Examples.

[3] Hernández-González J., Capdevila J., Cerquides J. (2019). Variational Importance Sampling: Initial Findings. Artificial Intelligence Research and Development: Proceedings of the 22nd International Conference of the Catalan Association for Artificial Intelligence.

[4] Koller D., Friedman N. (2009). Probabilistic Graphical Models: Principles and Techniques.

[5] Owen A., Zhou Y. (2000). Safe and Effective Importance Sampling. J. Am. Stat. Assoc..

[6] Basseville M. (2013). Divergence measures for statistical data processing—An annotated bibliography. Signal Process..

[7] Zhu H., Rohwer R. (1997). Measurements of Generalisation Based on Information Geometry. Mathematics of Neural Networks: Models, Algorithms and Applications.

[8] Murphy K.P. (2012). Machine Learning: A Probabilistic Perspective.

[9] Minka T. (2005). Divergence Measures and Message Passing.

[10] Hesterberg T. (1995). Weighted Average Importance Sampling and Defensive Mixture Distributions. Technometrics.

[11] Elvira V., Martino L., Luengo D., Bugallo M.F. (2015). Efficient Multiple Importance Sampling Estimators. IEEE Signal Process. Lett..

[12] Regli J.B., Silva R. (2018). Alpha-Beta Divergence For Variational Inference. arXiv.

[13] Wang D., Liu H., Liu Q. Variational Inference with Tail-Adaptive f-Divergence. https://papers.nips.cc/paper/2018/hash/1cd138d0499a68f4bb72bee04bbec2d7-Abstract.html.

[14] Erven T.V., Harremos P. (2014). Rényi Divergence and Kullback-Leibler Divergence. IEEE Trans. Inf. Theory.

[15] Li Y., Turner R.E. Rényi Divergence Variational Inference. https://proceedings.neurips.cc/paper/1992/file/7750ca3559e5b8e1f44210283368fc16-Paper.pdf.

